# Protection against Multiple Influenza A Virus Strains Induced by Candidate Recombinant Vaccine Based on Heterologous M2e Peptides Linked to Flagellin

**DOI:** 10.1371/journal.pone.0119520

**Published:** 2015-03-23

**Authors:** Liudmila A. Stepanova, Roman Y. Kotlyarov, Anna A. Kovaleva, Marina V. Potapchuk, Alexandr V. Korotkov, Mariia V. Sergeeva, Marina A. Kasianenko, Victor V. Kuprianov, Nikolai V. Ravin, Liudmila M. Tsybalova, Konstantin G. Skryabin, Oleg I. Kiselev

**Affiliations:** 1 Department of Influenza Vaccines, Research Institute of Influenza, Ministry of Health of the Russian Federation, St. Petersburg, Russia; 2 Centre “Bioengineering”, Russian Academy of Sciences, Moscow, Russia; 3 GenNanotech Ltd, St. Petersburg, Russia; Georgia State University, UNITED STATES

## Abstract

Matrix 2 protein ectodomain (M2e) is considered a promising candidate for a broadly protective influenza vaccine. M2e-based vaccines against human influenza A provide only partial protection against avian influenza viruses because of differences in the M2e sequences. In this work, we evaluated the possibility of obtaining equal protection and immune response by using recombinant protein on the basis of flagellin as a carrier of the M2e peptides of human and avian influenza A viruses. Recombinant protein was generated by the fusion of two tandem copies of consensus M2e sequence from human influenza A and two copies of M2e from avian A/H5N1 viruses to flagellin (Flg-2M2eh2M2ek). Intranasal immunisation of Balb/c mice with recombinant protein significantly elicited anti-M2e IgG in serum, IgG and sIgA in BAL. Antibodies induced by the fusion protein Flg-2M2eh2M2ek bound efficiently to synthetic peptides corresponding to the human consensus M2e sequence as well as to the M2e sequence of A/Chicken/Kurgan/05/05 RG (H5N1) and recognised native M2e epitopes exposed on the surface of the MDCK cells infected with A/PR/8/34 (H1N1) and A/Chicken/Kurgan/05/05 RG (H5N1) to an equal degree. Immunisation led to both anti-M2e IgG1 and IgG2a response with IgG1 prevalence. We observed a significant intracellular production of IL-4, but not IFN-γ, by CD4+ T-cells in spleen of mice following immunisation with Flg-2M2eh2M2ek. Immunisation with the Flg-2M2eh2M2ek fusion protein provided similar protection from lethal challenge with human influenza A viruses (H1N1, H3N2) and avian influenza virus (H5N1). Immunised mice experienced significantly less weight loss and decreased lung viral titres compared to control mice. The data obtained show the potential for the development of an M2e-flagellin candidate influenza vaccine with broad spectrum protection against influenza A viruses of various origins.

## Introduction

Conserved proteins of influenza A virus (M2, HA2, M1, NP) are the target antigens for development of “universal” vaccines against human influenza A viruses of a particular subtype as well as against influenza viruses of distinct origin including potentially pandemic avian ones.

Influenza М2 is a small, 97 amino acid (aa) protein that forms ion channels in the viral membrane. By regulating pH level, it provides the uncoating of viral particles in endosomes and prevents premature conformational rearrangement of newly synthesised haemagglutinin during transport to the cell surface by equilibrating the pH of the trans-Golgi network [1]. The tetramer М2 protein is expressed on viral surface in low quantities, but is abundantly presented on the plasma membrane of infected cells [2]. The М2 protein ectodomain (М2е)—is a short 24 aa length peptide that is highly conserved and thus presents an appropriate target for “universal” influenza vaccine development [3, 4]. Previous studies have shown that the immunogenicity of native М2е is poor, but it can be increased by using multimeric forms of M2e, the fusion of M2e to highly immunogenic carriers or application with adjuvants [5–14].

The flagellin protein presents the appropriate platform for the development of recombinant vaccines against various pathogens of viral and bacterial origin. As the major structural protein of Gram-negative bacteria, flagellin exhibits strong adjuvant properties when administered together with foreign antigens by parenteral, mucosal or subcutaneous routes [15–22]. Flagellin С- and N-terminal conserved regions bind leucine-rich repeats of Toll-like receptor 5 (TLR5) [23–25], thus activating the effectors of innate immunity [26, 27]. The ability of flagellin to serve as a platform and an adjuvant for vaccine development at the same time was demonstrated for various model infections including influenza [9, 28–35], *Yersinia pestis* [17], West Nile virus [18], *Pseudomonas aeruginosa* [22], *Schistosoma mansoni* [15], vaccinia virus [19, 36], *Campylobacter jejuni* [16], *Salmonella typhimurium* [37–39], *Salmonella paratyphi A* [40], and *Streptococcus pneumonia* [41]. These studies showed that heterologic peptides could be linked to the N- and C-terminus of flagellin or be inserted into the hypervariable region without disturbing the ability of flagellin to bind TLR5 [16, 18, 29, 30, 37]. Furthermore, flagellin was demonstrated to provide a strong immune response to low immunogenic proteins and peptides. [9, 36]

The adjuvant effect of flagellin is shown to be due to the induction of cytokine production by non-lymphoid cells, the increased accumulation of Т- and В-cells in lymph nodes, and the activation of tlr5+CD11c+ cells. The ability of flagellin to bind with high affinity to TLR5 on CD11c+ antigen-presenting cells (APC) explains the increased potential of fused antigen-flagellin proteins to stimulate CD4+ T-cell dependent humoral immune response in comparison with unfused antigen + flagellin [17, 21, 36, 42].

TLR5 is abundantly expressed in lung tissues [43, 44] and plays an important role in protection against respiratory pathogens, thereby increasing the benefit of flagellin as an effective mucosal adjuvant. The intranasal route of antigen administration mimics natural infection and induces both local and systemic immune responses. Local immunity is mediated by secretory immunoglobulin A (sIgA), the multimeric form of which has an effective antiviral activity. Secretory IgA can form immune complexes on the mucosal surface as well as inside infected epithelial cells without damaging the tissue, thereby decreasing viral attachment and penetration into the cells, and aborting viral replication and the formation of viral progeny in the infected cell [45, 46]. Moreover, it has been demonstrated that IgA plays an important role in the functioning of APC, developing antiviral memory T-cells and the formation of subtype-specific immunity [45]. Another advantage of mucosal vaccines is low reactogenicity and the minimal risk of accidental infection with foreign pathogens in comparison with intramuscular preparations.

In this study, we investigated the possibility of developing an M2e-based candidate vaccine that is capable of forming protective immune response simultaneously against human and avian influenza A viruses. We designed the recombinant protein (Flg-2M2eh2M2ek), where 4 tandem copies of M2e (2 copies of M2e consensus sequence of human influenza A viruses (M2eh) and 2 copies of M2e sequence of avian influenza virus A/H5N1 (M2ek)) were linked to the C-terminus of a full length flagellin. We demonstrated that intranasal immunisation of BALB/c mice with Flg-2M2eh2M2ek resulted in equal antibody response to M2eh and M2ek in serum and broncho-alveolar lavage (BAL). In addition, induced anti-M2e antibodies recognised native M2e epitopes exposed on the surface of the MDCK cells infected with A/PR/8/34 (H1N1) and A/Kurgan/5/05 RG (H5N1). We also observed significant intracellular production of IL-4, but not IFN-γ, by CD4+ T-cells in spleen of mice following immunisation with Flg-2M2eh2M2ek and the prevalence of anti-M2e IgG1. We conclude that immunisation with Flg-2M2eh2M2ek fusion protein protects mice in a similar way from a lethal challenge with human influenza A viruses (H1N1, H2N2, H3N2) and avian influenza virus (H5N1), and results in significantly less weight loss and decreased lung viral titres compared to control mice.

## Materials and Methods

### Construction of expression vectors

Plasmid pQE30 (Qiagen) was used to construct a vector for the expression of fusion protein comprising four copies of the M2e peptide and flagellin of *Salmonella typhimurium* (Flg). In the first step, the sequence encoding human consensus M2e peptide (SLLTEVETPIRNEWGCRCNDSSD), M2eh, was obtained by the annealing of two complementary synthetic oligonucleotides, M2hF (GATCCAGTCTGCTTACGGAGGTTGAAACCCCAATTCGCAACGAGTGGGGTTGCCGTTGCAATGATAGCAGTGACCTGCA) and M2hR (GGTCACTGCTATCATTGCAACGGCAACCCCACTCGTTGCGAATTGGGGTTTCAACCTCCGTAAGCAGACTG), and cloned at the BamHI and PstI sites of pQE30 producing the plasmid pQE30_M2h. Then, an artificial sequence encoding two copies of the M2e peptide of avian influenza virus strain A/Chicken/Kurgan/05/2005 (SLLTEVETPTRNEWECRCSDSSD), M2ek, and one copy of M2eh, arranged as M2ek-M2eh-M2ek, were commercially synthesised (Evrogen, Russia). Cloning of this synthetic gene at the PstI and KpnI sites of pQE30_M2h resulted in construction of the expression vector pQE30_2M2eh2M2ek. Finally, flagellin gene *fljB* of *S*. *typhimurium* was obtained by PCR with the primers FlgBH_F (atggatccGCACAAGTAATCAACACTAACAGTCTGT) and FlgBH_R (atggatccACGTAACAGAGACAGCACGTTCTGC) and cloned at the BamHI site of pQE30_2M2eh2M2ek, resulting in construction of the expression vector pQE30_Flg2M2eh2M2ek. This vector allowed the production of recombinant protein Flg-2M2eh2M2ek carrying N-terminal histidine tag and consisting of flagellin and four copies of M2e peptide (arranged as Flg-M2eh-M2ek-M2eh-M2ek).

### Expression and purification of recombinant protein Flg-2M2eh2M2ek

For the expression of recombinant protein, the corresponding vector was introduced into *E*. *coli* strain DLT1270. The strain was grown in LB until the midpoint of the logarithmic growth phase (OD_600_ = 0.5) at 37°C, then IPTG was added to 1 mM endpoint concentration and the culture was grown for a further 12–14 h at 28°C. After induction, the bacteria were collected by centrifugation and resuspended in 20mM phosphate buffer, pH 7.2. This suspension was supplemented with 1mM PMSF and treated with 1 mg/ml lysozyme for 15min on ice followed by sonication. The suspension was then centrifuged at 13,000 g for 5 min. The supernatant was applied to Ni-NTA-resin equilibrated with 20mM phosphate buffer, pH 8.0, containing 5 mM imidazole and incubated for 60 min. Following binding of the target protein, the resin was washed with 20mM phosphate buffer, pH 8.0, containing 20 mM imidazole. The recombinant protein Flg-2M2eh2M2ek was eluted with 20mM phosphate buffer, pH 8.0, containing 0.5 M imidazole and dialysed against 10mM phosphate buffer, pH 7.2 (PBS).

### Protein ELISA

The identity and integrity of recombinant protein was determined in ELISA [9]. To confirm identity and epitope display, ELISA plates were coated overnight at 4°C with serial dilutions of Flg-2M2eh2M2ek, Flg-HA2 or synthetic M2e peptide in PBS. As a control protein, we used Flg-HA2 (recombinant protein in which 68 aa of A/H2N2 HA2 was fused to C-terminus of Flg). The plates were blocked with PBS containing 5% foetal calf serum (FCS) (300 μl/well) for 1 hour at room temperature. The plates were probed with rabbit polyclonal antibodies specific for flagellin (ab93713, Abcam, UK) or M2e (ab5416, Abcam, UK) overnight at 4°C. HRPO-labelled rat anti-mouse IgG antibodies (Invitrogen, USA) diluted in PBS with 5% FCS were added (100 μl/well) to the plates. Further incubation was performed at room temperature for 1 hour. TMB substrate Reagent Set (BD Pharmingen, USA) was used according to the manufacturer’s recommendations. The reaction was monitored by measuring the OD at 450 nm on a microplate spectrophotometer (Bio Rad, USA).

To confirm integrity of the recombinant protein, a two-site sandwich ELISA was performed. ELISA plates were coated with flagellin-specific ab93713. After blocking, serial dilutions of the test proteins Flg-2M2eh2M2ek and Flg-HA2 were added. Plates were probed with M2e-specific mAb 14C2. Incubations and development were performed as described above.

### Western blotting

The proteins were separated in an 8–20% gradient gel by reducing SDS-PAGE and electro-transferred to a nitrocellulose membrane (Bio-Rad, USA). The membrane was blocked with 3% BSA overnight at room temperature and protein bands were detected by staining membrane with rabbit polyclonal antibodies specific for bacterial Flagellin (ab93713, Abcam, UK) or mouse anti-M2e monoclonal antibody 14C2 (ab5416, Abcam, UK). The membrane was incubated for 1h at room temperature with antibodies diluted in PBS with 0.1% Tween-20 (PBST) and 3% BSA and then washed with PBST. Bands were visualised by staining the membrane for 1 hour at room temperature with the peroxidase-labelled secondary antibodies goat anti-rabbit IgG (Invitrogen, USA) or goat anti-mouse IgG (Abcam, UK) and subsequent incubation in TMB Immnublot solution (Invitrogen, USA) for 5 min.

### Endotoxin measurement

Endotoxin levels were determined using LAL-test (Charles River Endosave) as directed by the manufacturer.

### Ethics Statement

The study was carried out in strict accordance with the Russian Guidelines for the Care and Use of Laboratory Animals (1977). The protocol was approved by the Committee for Ethics of Animal Experimentation of the Research Institute of Influenza (Permit Number: 01213). All efforts were made to minimise animal suffering. Mice were housed in cages provisioned with water and standard food and monitored daily for health and condition. More than 25% body weight loss was used as a criterion for early euthanasia. The animals were euthanised by CO_2_ inhalation for 5 minutes. After final monitoring (14 day post-challenge), all of the surviving mice were humanely euthanised using CO_2_ inhalation for 5 minutes.

### Mouse immunisation

Female BALB/c mice (16–18g) were purchased from the Institute of Animal Care (Pushchino, Russia). Mice were immunised intranasally (i.n.) on days 0 (primary vaccination), 14 (first boost), 28 (second boost) with 50 μg/0.1ml of Flg-2M2eh2M2ek (7.5 μg of M2e) under inhalation anaesthesia (2–3% isoflurane mixed with 30% oxygen (O_2_) and 70% nitrous oxide (N_2_O)). Control mice were injected i.n. with 0.1 ml of PBS. Blood samples from the ventral vein of 5 mice were collected 2 weeks after the first boost. Blood samples, BAL from 5 mice (sacrificed by CO_2_-box for euthanasia—Vet Tech Solutions) were collected 2 weeks post-second boost. Mice were dissected to expose the trachea and then an IV catheter (BD Bioscience) was inserted into a small nick in the trachea. BAL samples were collected by 2-fold flushing the airway compartment with 500μl of PBS. The samples were stored at −20°C until use.

### Synthetic peptides

The following peptides were tested in the ELISA experiments:

M2ek SLLTEVETP**T**RNEW**E**CRC**S**DSSD (M2e of A/Chicken/Kurgan/05/05 H5N1)

M2eh SLLTEVETP**I**RNEW**G**CRC**N**DSSD (consensus M2e of human influenza A viruses)

Residues that differ between the sequences are displayed in bold font and underlined.

### Antibody detection in the sera and BAL

M2e-specific IgG and IgA levels were determined by ELISA in 96-well microtitre plates (“Greiner”, Germany) coated overnight at 4°C with the M2e peptides (100μl/well) in PBS (5 μg/ml). The plates were blocked with PBS containing 5% FCS (300 μl/well) for 1 hour at room temperature. Then, the plates were washed 3 times in PBST. Sera or BAL were serially diluted by PBS with 5% FCS. The diluted samples were added in volumes of 100μl/well and the plates were incubated for 1 hour at room temperature. The plates were washed 3 times in PBST. HRP-labelled goat anti-mouse mAb IgG (Invitrogen, USA), goat anti-mouse mAb IgG1 (Invitrogen, USA), IgG2a (Invitrogen, USA), and IgA (Invitrogen, USA) were diluted in PBS with 5% FCS and added (100 μl/well) into the plates. Further incubation was performed for 1 hour at room temperature. Final washing was carried out 4 times in PBST. The plate content was developed with the TMB substrate Reagent Set (BD Pharmingen, USA) according to the manufacturer’s recommendations. The reaction was stopped using 50 μl 2M H_2_SO_4_ and OD_450_ was measured on a microplate spectrophotometer (Bio Rad, USA). ELISA endpoint titres were defined as a reciprocal of the highest dilution yielding an OD_450_ value 2 times above the mean value of negative control wells.

### MDCK whole cell ELISA

Sera were tested for reactivity with MDCK NBL-2 (Madin-Darby Canine Kidney) epithelial cells (received from the Russian Collection of Vertebrate Cell Cultures, Institute of Cytology, RAS, Saint-Petersburg, Russia) infected by influenza A, as reported previously [9]. The MDCK cells were cultured in DMEM medium (Paneco, Russia) containing 5% FCS until they became confluent. Then, the cells were incubated overnight at 37°C with 10^6^ 50% egg infective dose (EID_50_) of influenza virus A/PR/8/34 (H1N1), 10^5^ EID_50_ of influenza virus A/Kurgan/05/05 RG (H5N1) or with the medium alone (uninfected control). Subsequently, the plates were washed with PBS and fixed by 10% formaldehyde for 10 min at room temperature. After that, the plates were washed 3 times with PBS and blocked with PBS+5% FCS for 1 hour at room temperature. Serial dilutions of the sera were added to the cells and stored for 1 hour. Wells were washed and incubated with HRPO-labelled goat anti-mouse IgG mAb (Invitrogen, USA) for 1 hour at room temperature, followed by incubation with the TMB substrate Reagent Set (BD Bioscience, USA) for a further 15 min at room temperature. The reaction was stopped with 50 μl 2M H_2_SO_4_ and OD_450_ was measured on a microplate spectrophotometer (Bio Rad, USA). The data reflected the mean ΔOD (infected-uninfected cells) of triplicate wells per sample.

### Isolation of Spleen Cells

On day 14 post-second boost, 3 mice from immunised and control groups were sacrificed by CO_2_-box for euthanasia (Vet Tech Solutions). Spleens were aseptically removed and splenocytes were harvested after homogenisation using Medimachine (BD Biosciences, USA) and the removal of debris by filtration through a 70 μm syringe filcons (BD Biosciences, USA). Red blood cells (RBCs) were lysed using an ACK buffer (0.15M NH_4_Cl, 1.0М KHCO_3,_ 0.1 mМ Na_2_EDTA, pH 7.2–7.4) and splenocytes were washed several times with RPMI-1640 complete medium containing 10% FCS, 2 mM L-glutamine, 100 IU/ml penicillin, and 100 mg/ml streptomycin. Cell viability was determined by trypan blue dye (0.4% w/v) exclusion.

### Splenocyte proliferation assay

The proliferative assay was performed as described [47]. Spleens of immunised and control mice were dissected on day 14 after the last immunisation and the proliferative response to M2eh peptide was tested. The cells were cultured in 96-well plates (Orange scientific, Belgium) using 50μl triplicates of splenocyte suspensions (1х10^6^ cells/ml) in complete RPMI-1640 medium with 7.5% FCS. Splenocytes were stimulated with 10 μg/100μl of peptide M2eh or 1 μg/ml ConA (Sigma, USA) as a positive control. The plates were incubated in 5% СО_2_ at 37°С for 72h. After incubation, 100μl of stock MTT (Sigma, USA) solution (5μg/ml) was added to all wells and plates were incubated at 37°С for 4h. Acid-isopropanol (100 μl of 0.04N HCl in isopropanol) was added to all wells and thoroughly dissolved the dark blue crystals. After a few minutes at room temperature, OD_535_ was measured on the microplate reader. The index of stimulation (IS) was calculated using the following equation: OD of M2e-treated cells/OD of untreated cells.

### Intracellular Cytokine Staining (ICS) assay

Multi-parameter flow cytometry was performed in accordance with the BD Protocol. Briefly, splenocytes were harvested at day 14 post-immunisation, and 2x10^6^ splenocytes were stimulated for 6 h at 37°C with 10 μg of M2eh peptide in the presence of 1μg/ml of Brefeldin A (BD Bioscience, USA). The cells were then washed and Fc receptors were blocked using CD16/CD32 antibodies (Mouse BD Fc Block, BD Pharmingen, USA) and stained with anti-CD3а-FITC (BD Pharmingen, USA) and anti-CD4-APC (BD Pharmingen, USA) at 4°C for 30 min. Cells were permeabilised using BD Cytofix/Cytoperm Plus (BD Bioscience, USA) protocols and stained with anti-IL-4-PE (BD Pharmingen, USA) or anti-IFN-γ-PE (BD Pharmingen, USA). Sample acquisition (50,000 events were collected) was performed with a BD FACS Canto II flow cytometer (Becton Dickinson, USA) and analysed using BD FACS Diva version 6.1.3 (BD Bioscience, USA).

### Influenza virus challenge of mice

The mice (n = 10/group) were challenged i.n. with 5 50%-lethal doses (5LD_50_) of influenza viruses A/PR/8/34 (H1N1), A/Aichi2/68 (H3N2), A/Chicken/Kurgan/05/05 RG (H5N1) 2 weeks after the final immunisation. Mice were anaesthetised under inhalation anaesthesia (2–3% isoflurane mixed with 30% oxygen (O_2_) and 70% nitrous oxide (N_2_O)). Influenza viruses A/PR/8/34 (H1N1), A/Aichi/2/68 (H3N2), A/Chicken/Kurgan/05/05 RG (H5N1) were received from the Collection of Influenza and ARI Viruses at the Research Institute of Influenza. Influenza A/Chicken/Kurgan/05/05 RG is avirulent strain obtained by reverse genetics in the Research Institute of influenza. 7 genes (all except the HA gene) of A/Chicken/Kurgan/05/05 RG are identical to highly pathogenic wild-type virus A/Chicken/Kurgan/05/2005 (H5N1) (GenBank, accession numbers: DQ449632-DQ449639). The HA gene of A/Chicken/Kurgan/05/05 RG was derived from wild-type by modifying the HA cleavage site: polybasic amino acid sequence was changed by TETR/GLF sequence, typical for avirulent H5 viruses. Experimental work with A/Chicken/Kurgan/05/05 RG was carried in BSL2+ (enhanced) facility. In strong agreement with national requirements all precautions were made to minimize the risk associated with H5N1 viruses. The indicated BSL2+ facility is separated from the other lab, equipped with biological safety cabinet, it has controlled ventilating system with HEPA-filtered air exhaust and only work with A/H5N1 influenza viruses is carried inside (no other influenza subtypes). A/Chicken/Kurgan/05/05 RG (H5N1) challenge experiments with animals were carried in BSL3 vivarium. Influenza stain A/Aichi/1/68 (H3N2) used for the challenge experiment was a mouse-adapted virus, obtained in the Research Institute of influenza by serial mouse/egg passages of wild-type origin. The mouse-adapted variant has retained the antigenic properties of wild-type origin but acquired the ability to lethally infect mice, its M2, NA aa sequence and HA remained identical to wild-type origin (S1A,B Fig., S1 Table). Mice administered with PBS were challenged as a negative control. The animals were monitored for survival and weight loss daily for 2 weeks.

### Lung virus titres

The mice (n = 5/group) were challenged i.n. with 1LD_50_ of influenza viruses A/PR/8/34 (H1N1), 1LD_50_ A/Singapore/1/57 (H2N2), 5LD_50_ of A/Aichi/2/68 (H3N2) or 5LD_50_ of A/Chicken/Kurgan/05/05 RG (H5N1) two weeks after the final immunisation. Experimental work with the A/Singapore/1/57 (H2N2) virus was carried out using BSL3 conditions. Five mice from each group were sacrificed by CO_2_-box for euthanasia (Vet Tech Solutions) on day 6 post-infection, the lungs were removed aseptically, homogenised in 2.7 ml PBS using a Tissue Lyser II homogeniser (Qiagen, USA) to achieve 10% (w/v) suspensions of lung and centrifuged (15 min, 400g, 4°C) to remove cellular debris before storage at −20°C. In 96-well cell-culture plates, ten-fold serial dilutions of samples were added to monolayers of MDCK (in serum-free medium with 2μg/ml of TPCK-trypsin (Sigma)) in quadruplicate and incubated as above for 72h. Viral cytopathic effect was observed daily and viral titre was determined by HA test with 0.5% chicken erythrocytes. The viral titre was calculated by the Reed and Mench method and expressed as lg 50% tissue culture infectious dose (TCID_50_).

### Statistical analysis

The differences between antibody levels, index of proliferation, percent of CD3^+^CD4^+^ IL-2 and IFN-γ producing cells, and viral titres in lung suspensions were evaluated by the Mann-Whitney U-test. Significant differences in survival among mouse groups were analysed by the Montel-Cox test, while the difference in weight loss was evaluated by the Wilcoxon test. If a p value was less than 0.05, the difference was considered significant.

## Results

### Design, expression and purification of Flg-2M2eh2M2ek fusion protein

**Fig 1:**
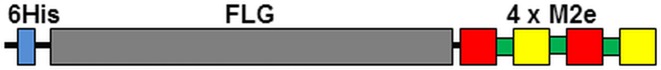
Structure of recombinant protein Flg-2M2eh2M2ek. Blue box, 6 histidine tag; grey box, flagellin of *S*. *typhimurium*; red boxes, human “consensus” M2e peptide; yellow boxes, M2e peptide of avian influenza strain А/Chicken/Kurgan/5/05 (H5N1); green boxes, flexible glycine-rich linkers. Sizes of boxes are not drawn to scale.

A fusion protein Flg-2M2eh2M2ek containing two copies of human consensus M2e sequence SLLTEVETPIRNEWGCRCNDSSDP (M2eh) and two copies of the avian influenza virus strain A/Chicken/Kurgan/05/2005 M2e peptide SLLTEVETPTRNEWECRCSDSSD (M2ek) fused to the C-terminus of *S*. *typhimurium* FljB was designed (Fig. 1). In order to facilitate folding of the hybrid protein, flexible glycine-rich linkers (GGGSG) were inserted between sequences of M2e peptides. The corresponding hybrid gene was cloned into the plasmid vector pQE30 and expressed in *E*. *coli*. The recombinant fusion protein carrying an N-terminal 6-histidine tag was purified using standard nickel affinity chromatography.

**Fig 2:**
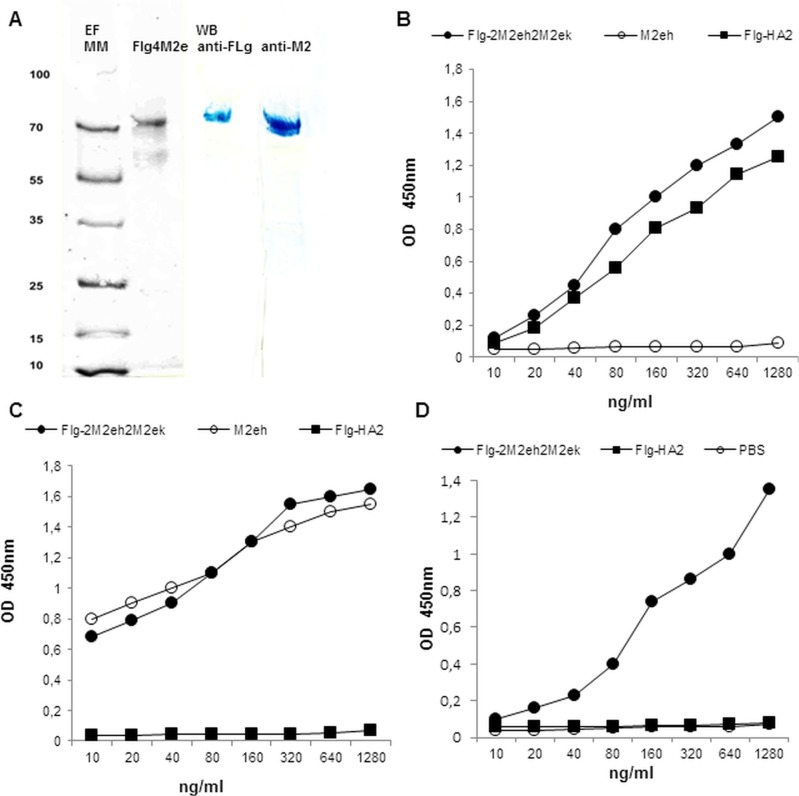
Antigenicity and integrity of Flg-2M2eh2M2ek. (A) SDS-PAGE (EF) Coomassie brilliant blue staining and Western blotting (WB) analysis of Flg-2M2eh2M2ek (Flg4M2e) by anti-Flg mAb 93713 and anti-M2e mAb 14C2, immunostaining with HRP-conjugated second antibodies and TMB substrate. Positions of molecular weight markers (MM) are indicated. (B) Recombinant Flg-2M2eh2M2ek, Flg-HA2 and a synthetic 24 amino acid peptide corresponding to human M2e consensus were coated on ELISA plates and then probed with monoclonal antibody ab93713 specific for flagellin (B) or monoclonal antibody 14C2 specific for M2e (C). Protein integrity was measured in a sandwich ELISA (D). Recombinant proteins Flg-2M2eh2M2ek and Flg-HA2 were captured on plates coated with mAb 93713 specific for flagellin, and detected with mAb 14C2 specific for M2e.

The purity of the Flg-2M2eh2M2ek recombinant protein was evaluated by SDS-PAGE which indicated a single band with a molecular weight of approximately 66 kDa and 98% purity (Fig. 2A). The identity and integrity of the recombinant protein were estimated by Western blot analysis (Fig. 2A) and ELISA with the antibodies specific to flagellin (Fig. 2B) and 14C2 mAb to M2e (Fig. 2C). The results confirmed the presence of M2e and flagellin in the purified protein. The integrity of recombinant protein was confirmed by sandwich ELISA. The results in Fig. 2D demonstrate that the Flg-2M2eh2M2ek protein is intact, with both M2e epitopes and flagellin being present in the same molecule. As mAb 14C2 has been shown to recognise protective epitopes in M2 [48, 49], the current results demonstrate that the protective epitope of M2e is presented in Flg-2M2eh2M2ek protein.

Evaluation of the Flg-2M2eh2M2ek fusion protein in LAL assays demonstrated that the final preparation contained an endotoxin level of 0.9 EU/μg (data not shown).

### M2e specific antibody response in serum

**Fig 3:**
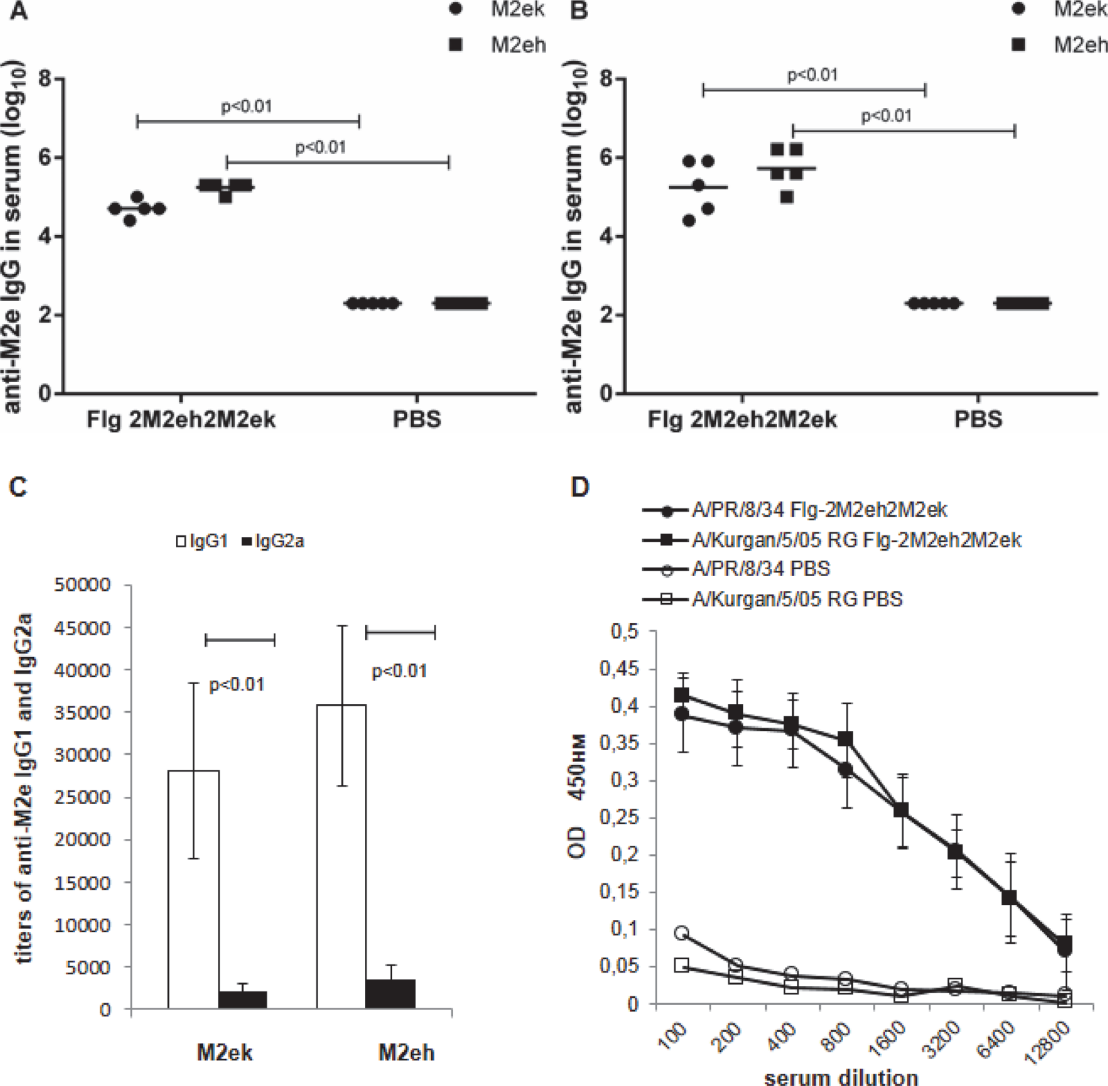
Anti-M2e antibody response in serum. BALB/c mice (n = 5/group) were immunised i.n. with 50 μg of Flg-2M2eh2M2ek recombinant protein on days 0, 14, 28. Mice of control group were administered with PBS. Two weeks post-first (A) and second boosts (B) M2e-specific IgG responses were evaluated by ELISA to M2eh and M2ek synthetic peptides. Horizontal bars indicate mean titres among 5 mice per group. (C) Anti-M2e IgG subclasses tested against M2ek and M2eh in serum 2 weeks post-second boost were determined by ELISA. Statistically significant differences between IgG1 and IgG2a levels: p<0.01. Statistical significance was determined using Mann-Whitney U-test. The P values between immunised and control group are indicated. (D) Recognition of influenza-infected MDCK cells by antisera from Flg-2M2eh2M2ek immunised mice. PBS line refers to control mice group, immunised i.n. with PBS. Two weeks post-second boost sera were tested for reactivity with non-infected and infected with A/PR/8/34 (H1N1) and A/Chicken/Kurgan/05/05 RG (H5N1) MDCK cells *in vitro*. Data represent the ΔOD_450_ (infected—uninfected cells).

The immunogenicity of fusion protein Flg-2M2eh2M2ek was examined in Balb/c mice immunised i.n. on the days 0, 14, 28. On the days 28 and 42 mice were bled and sera of 5 mice were tested individually for anti-M2e IgG, IgG1 and IgG2a by ELISA. A strong specific immune response was induced post-first boost and further increased post-second boost (Fig. 3A, B). Induced antibodies efficiently bound to synthetic M2eh and M2ek peptides, corresponding to M2e sequences in the recombinant protein. No significant differences were found in levels of anti-M2eh and anti-M2ek IgG (p>0.05). We also examined the profile of M2e-specific IgG subclass after the second boost immunisation of mice with Flg-2M2eh2M2ek (Fig. 3C). Intranasal immunisation with fusion protein based on flagellin led to the formation of both anti-M2e IgG1 and anti-M2e IgG2a. However, the level of serum IgG1 subclass was significantly higher than that of IgG2a (p<0.01) and the IgG1/IgG2a ratio was 12.6 for M2ek and 10.6 for M2eh.

In order to determine whether anti-M2e antibodies recognise native M2e, we used an MDCK whole cell ELISA. MDCK cells were grown in tissue culture plates. Then, they were infected with A/PR/8/34 (H1N1) or A/Chicken/Kurgan/05/05 RG (H5N1) influenza viruses, and fixed and incubated with serial dilutions of immune and non-immune sera. The sera taken from the mice immunised with Flg-2M2eh2M2ek (Fig. 3D) bound specifically and similarly to the MDCK cells infected with the influenza A/PR/8/34 (H1N1) and A/Chicken/Kurgan/05/05 RG (H5N1) viruses. We therefore conclude that the anti-M2e antibodies in immune serum are capable of recognising either the synthetic M2e peptides (M2eh and M2ek) or native M2e epitopes exposed on the surface of the MDCK cells infected with A/PR/8/34 (H1N1) and A/Chicken/Kurgan/5/05 RG (H5N1) to an equal degree.

### M2e-specific antibody response in BAL

**Fig 4:**
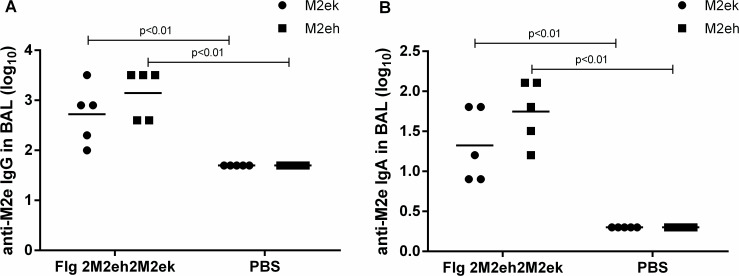
Anti-M2e antibody response in BAL. BALB/c mice (n = 5/group) were immunised i.n. with 50 μg of Flg-2M2eh2M2ek recombinant protein on days 0, 14, 28. Mice of control group were administered with PBS. Two weeks post-second boost M2e-specific IgG (A) and sIgA (B) responses were evaluated by ELISA to M2eh and M2ek synthetic peptides. Horizontal bars indicate mean titres among 5 mice per group. Statistical significance was determined using the Mann-Whitney U-test. The P values between immunised and control groups are indicated.

To investigate IgA and IgG responses in mucosal secretions, M2eh- and M2ek-specific IgA and IgG titres were determined in the BALs 2 week post-second boost in 5 mice from each group. As shown in Fig. 4AB, i.n. immunisation with Flg-2M2eh2M2ek fusion protein stimulated high levels of anti-M2e-specific IgG and sIgA in BAL. No significant differences were found between levels of anti-M2eh and anti-M2ek IgG and sIgA in BAL (p>0.05). Therefore, these results indicate that immunisation with recombinant protein with 2 copies of human M2e consensus and 2 copies of H5N1 M2e sequences lead to high yet equal levels of both anti-M2eh IgG, sIgA and anti-M2ek IgG, sIgA in BAL.

### M2e-specific T cell response in spleen

**Fig 5:**
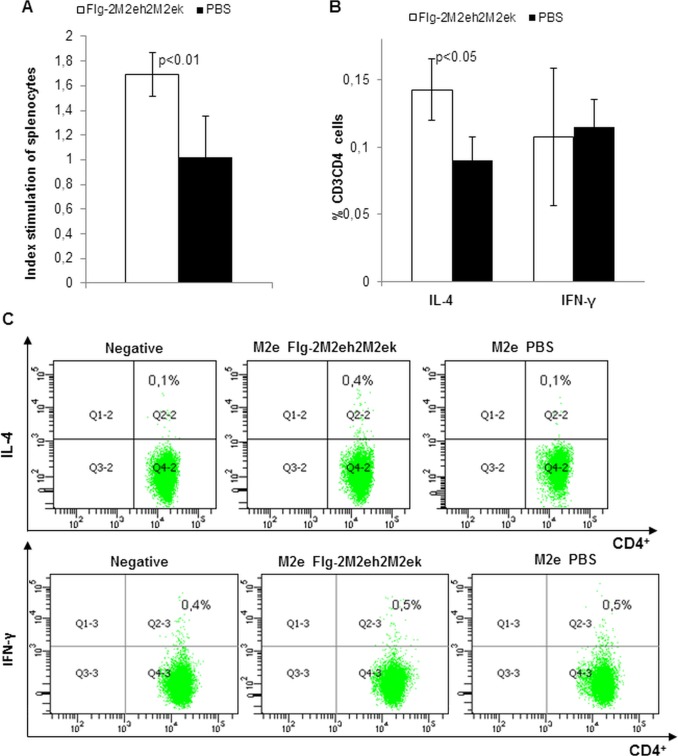
M2e specific T-cell response in spleen. BALB/c mice (n = 3/group) were immunised i.n. with 50 μg of Flg-2M2eh2M2ek recombinant protein on days 0, 14, 28. Splenocytes were isolated from 3 mice of each group at day 14 post-second boost and assayed for a M2e-stimulated proliferation (A) and M2e specific CD4^+^ T cell response (B). Data are presented as the mean±SEM. The index of stimulation (IS) was calculated using the following equation: OD of M2e-treated cells/OD of untreated cells. Statistical significance was determined using the Mann-Whitney U-test. The P values between immunised and control groups are indicated. (C) M2e-specific CD4^+^ T-cell response was determined by intracellular IL-4 and IFN-γ staining. Data are represented as representative density plots.

M2eh specific splenocytes proliferation was determined at day 14 post-second boost in 3 mice per group. As shown in Fig. 5A, the splenocyte proliferation level in those of the Flg-2M2eh2M2ek group was 1.7-fold higher than that of the unvaccinated animals in the PBS group (p<0.01).

To determine the phenotypic characteristics of T cell populations activated after immunisation with Flg-2M2eh2M2ek, we utilised multi-parameter intracellular flow cytometry staining (ICS) analysis to identify M2e specific T-cell responses. Splenocytes from 3 mice per group were stimulated by M2eh in the presence of brefeldin for 6 h. As shown in Fig. 5B, we observed a significant response following immunisation, and this profile was not polyfunctional (Fig. 5B,C); CD4+ T-cells secreted only IL-4 (p<0.05) but not IFN-γ (p>0.05). Overall, these results indicated that immunisation with Flg-2M2eh2M2ek induced an M2e-specific Th2 response.

### Immunisation with Flg-2M2eh2M2ek fusion protein protected mice against human and avian influenza viruses

To test whether immunisation with the Flg2M2ek2M2eh fusion protein could protect mice against human and avian influenza viruses, the groups of 12 mice immunised with 50μg (i.n., on days 0, 14 and 28) of recombinant fusion protein were challenged i.n. with 5LD_50_ of influenza viruses A/PR/8/34 (H1N1), А/Aichi/2/68 (H3N2) and А/Chicken/Kurgan/05/05 RG (H5N1) 2 weeks post-second boost. Mice administered with PBS were challenged as a control. After challenge, the animals were monitored daily for survival and weight loss over 2 weeks.

**Fig 6:**
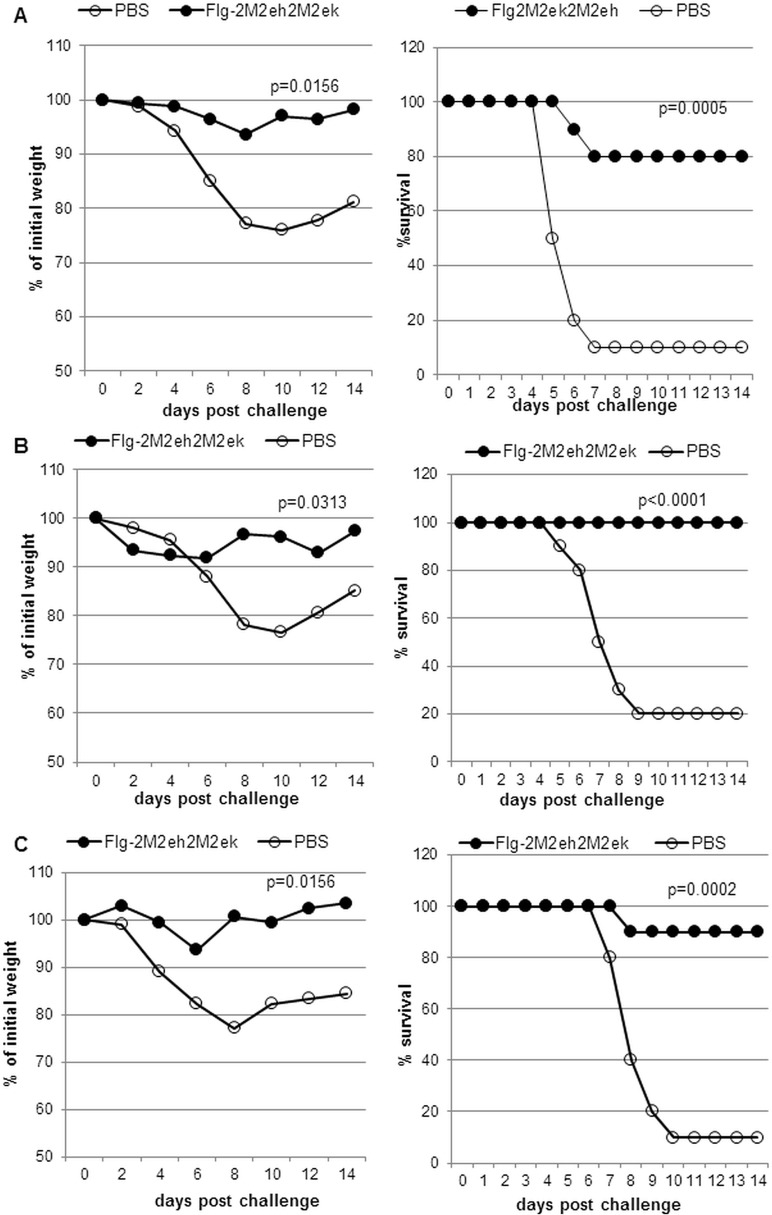
Efficacy of Flg-2M2eh2M2ek immunisation. Groups of 10 Balb/c mice were immunised with fusion protein Flg-2M2eh2M2ek. Two weeks post-second boost mice were challenged with (A) 5LD_50_ A/PR/8/34 (H1N1). Body weight (left; P = 0.0156, Wilcoxon test) and survival rate (right; P = 0.0005, Montel-Cox test) were monitored daily during 14 days; (B) with 5LD_50_ A/Aichi/2/68 (H3N2). Body weight (left; P = 0.0313, Wilcoxon test) and survival rate (right; P<0.0001, Montel-Cox test) were monitored daily during 14 days; (C) with 5LD_50_ A/Chicken/Kurgan/05/05 RG (H5N1). Body weight (left; P = 0.0156, Wilcoxon test) and survival rate (right; P = 0.0002, Montel-Cox test) were monitored daily during 14 days. The P values between immunised and control group are indicated.

As shown in Fig. 6A, 80% of mice treated with Flg2M2ek2M2eh survived upon the lethal challenge of A/PR/8/34 (H1N1) (p = 0.0005, Montel-Cox test) and experienced significantly less weight loss than the naïve animals (p = 0.0156, Wilcoxon test). The rate of survival among the mice after lethal challenge with 5LD_50_ of А/Aichi/2/68 H3N2 was 100%, while this index among the naïve mice was 20% (Fig. 6B), demonstrating enhanced survival (P<0.0001, Montel-Cox test) and less weight loss (P = 0.0313, Wilcoxon test) of the immunised mice compared to the control animals. The challenge of immunised mice with 5LD_50_ А/Chicken/Kurgan/05/05 RG H5N1 led to 90% survival (Fig. 6C). The difference in survival rates and weight loss between immunised groups and control animals was significant (P = 0.0002, Montel-Cox test; P = 0.0156, Wilcoxon test).

These results show that i.n. immunisation with the Flg-2M2eh2M2ek fusion protein protects mice against challenge with both human influenza A viruses (H1N1, H3N2) and avian influenza virus (H5N1).

### Immunisation with Flg-2M2eh2M2ek fusion protein significantly reduced influenza virus load in lungs

**Fig 7:**
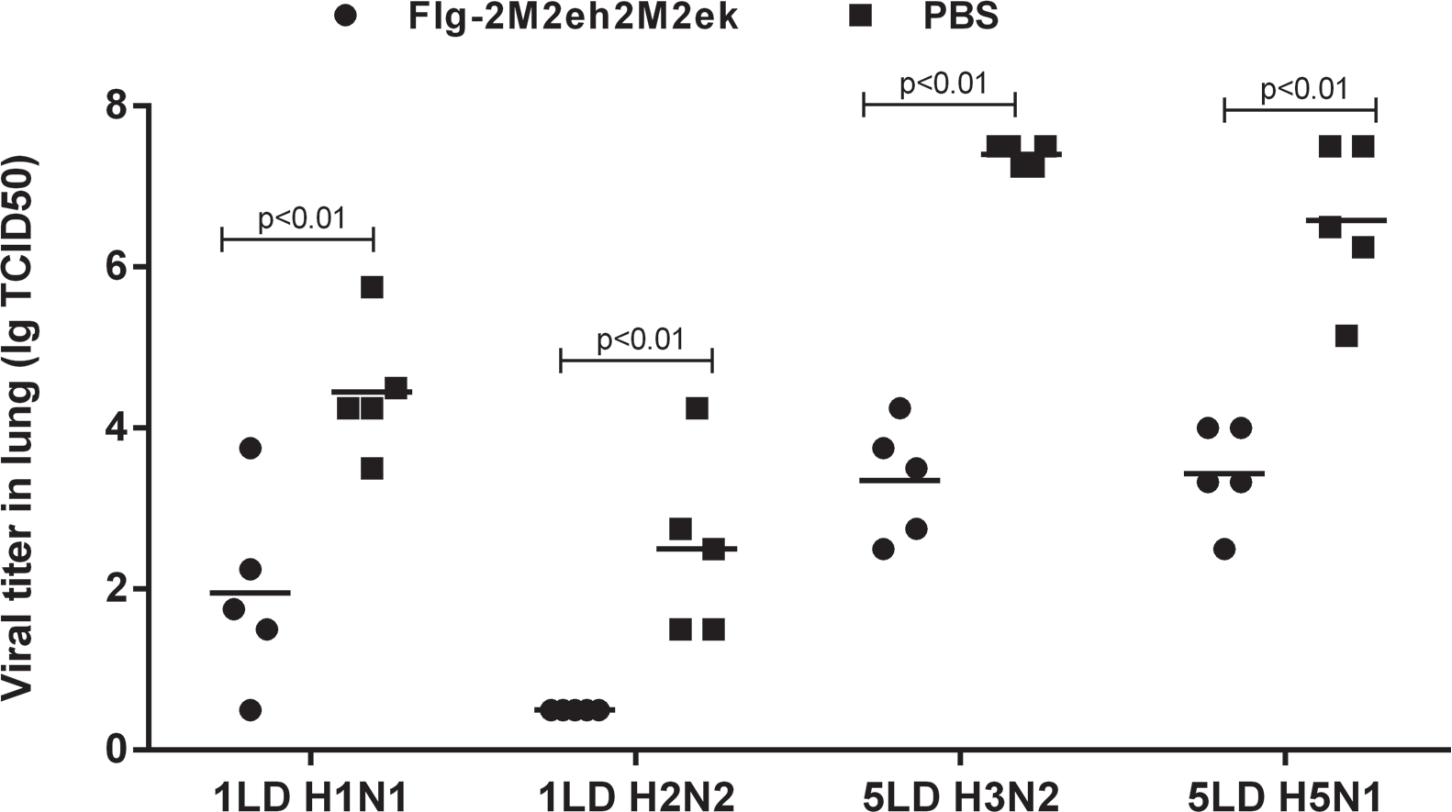
Detection of viral titre in mouse lung. Mice (n = 5/group) immunised with Flg-2M2eh2M2ek fusion peptide were i.n. challenged with 1LD_50_ А/PR/8/34 (H1N1), 1LD_50_ А/Singapore/1/57 (Н2N2), 5LD_50_ А/Aichi/2/68 (H3N2), 5LD_50_ А/Chicken/Kurgan/05/05 RG (H5N1) and viral titres were detected 6 days post-challenge. The data are expressed as lg TCID_50._ Horizontal bars indicate mean among 5 mice per group. The lower limit of detection is 0,5 lg TCID_50._ Statistical significance was determined using the Mann-Whitney U-test. The P values between immunised and control groups are indicated.

Two weeks after completion of the vaccination protocol, mice from the test and control groups were infected i.n. with 1LD_50_ of A/PR/8/34 (H1N1) and A/Singapore/1/57 (H2N2), 5LD_50_ of А/Aichi/2/68 (H3N2) and А/Chicken/Kurgan/05/05 RG (H5N1) influenza virus strains. Six days post-challenge, five mice from each group were sacrificed for the titration of residual lung virus. We observed a significant decrease in lung viral titres following challenge of immunised mice compared to naïve mice (Fig. 7). The Flg-2M2eh2M2ek immunisation decreased lung viral titres by 1.83, 2.0, 2.5 and 4.05 log_10_ upon challenge with А/Chicken/Kurgan/05/05 RG (H5N1), A/Singapore/1/57 (H2N2), A/PR/8/34 (H1N1) and А/Aichi/2/68 (H3N2), respectively.

## Discussion

The M2e sequence is well conserved among influenza A virus strains isolated from humans [5, 50, 51], but differs from influenza strains of animal origin, such as the avian influenza A virus H5N1 subtype that caused hundreds of illnesses and lethal outcomes, suggesting that a specific M2e-based vaccine against such strains would be required. The analysis of M2e sequenses from 716 influenza virus strains showed that region M2e 10–20 аa consistent with host restriction specificities of human (P**I**RN**E**W**G**C**R**C**N)**, avian species (P**T**RN**G**W**E**C**K**C**S)** and swine species (P**I**RN**G**W**E**C**R**C**N**) and protective mAb 8C6 against the EVET**PI**RN (aa 6–13) region of human influenza M2e weakly recognise avian M2e EVET**PT**RN from A/Netherlands/2003 (H7N7), A/ Vietnam/2004 (H5N1) and could not recognise avian M2e EVET**LT**RN from A/Kong Hong/1997 H5N1 [51]. So single mutation on M2e of some avian influenza strains weakened the binding of human M2e sequence specific mAb and double mutation completely eliminated such interaction. M2e of avian influenza virus A/H5N1 differs from the consensus M2e of human influenza A viruses by several aa. These differences are important and define the specificity of M2e-vaccines, as detected by only partial protection or protection failure post-heterologous challenge [52–55]. Recent studies showed that only four mutations in the haemagglutinin protein of the avian influenza virus A/H5N1 essentially changed virus receptor specificity and enhanced viral transmission in mammals [56]. Also, the reassortants between influenza virus A/H5N1 with modified HA and pandemic virus A/H1N1/pdm09 were able to be transmitted using the droplet method among polecats [57]. These experiments showed the possibility of virus adaptation from avian to human hosts; therefore, developing an M2e-based vaccine which protects from influenza viruses of distinct origin would be of interest.

Previously, it was shown [9] that a construct with four tandem copies of homologous M2e (human type M2e) fused to the bacterial flagellin was highly immunogenic and fully protected mice from influenza virus A/PR/8/34. Not long ago, a tandem repeat construct of heterologous M2e sequences (M2e5x) derived from human, swine, and avian origin influenza A viruses and incorporated in virus-like particles (M2e5x VLPs) in a membrane-anchored form was developed [13]. In that study, it was demonstrated that intramuscular immunisation of mice with M2e5x VLPs induced the broader reactivity of antibodies and improved cross-protection compared to the use of homologous M2e vaccine. So the multiple effect of M2e-vaccine with M2e of distinct origin is an important benefit in situations when a pandemic influenza virus comes into being and spreads quickly. Bearing in mind potential pandemic threat of virus types H5N1, H7N9 etc, necessary steps shall be taken: to create a structured panel of recombinant proteins with M2e of the said viruses and to develop respective producers of recombinant proteins.

We engineered a recombinant protein with tandem repeats of heterologous M2e sequences. The recombinant protein included the M2e of human influenza viruses (M2eh) and the avian influenza virus A/H5N1 (M2ek), which differed by 3 aa at positions 11, 16 and 20.

The recombinant protein Flg-2M2eh2M2ek with 4 copies of М2е consecutively linked to the C-terminus of full length flagellin (N-Flg-С-M2eh-M2ek-M2eh-M2ek) was designed. We demonstrated the ability of the fusion protein to induce high titres of serum and BAL anti-M2e IgG, binding to synthetic peptides M2eh and M2ek to a similar level. Our results showed that i.n. immunisation of Balb/c mice with Flg-2M2eh2M2ek protect immunised mice from lethal challenge with human (H1N1, H3N2) and avian (H5N1) influenza A viruses to an equal degree.

The upper respiratory tract contains plenty of APC, especially mucosal dendritic cells (DC) expressing TLR5 [58, 59]. Flagellin binds to TLR5 on the surface of APC thus activating the signals of innate immunity, cytokine secretion and regulation of other immune cells, and also increases antigen capture by DC and its subsequent presentation to CD4+ T-cells [60, 61]. Our results demonstrate that the fusion protein Flg-2M2eh2M2ek induces the formation of a strongly pronounced specific mucosal immune response. Also, titres of anti-М2е sIgA to linear epitopes of synthetic peptides M2eh and M2ek were similar. As reported earlier, M2e-based vaccines induce mucosal immunity when administered intranasally [8, 62, 63]. Mucosal IgA may bind intracellularly to M2e in infected cells, thereby restricting influenza virus replication [64]. In addition, the mechanism of anti-M2e sIgA protection may be mediated by the activation of effector cells through FcαμR in mice or CD86 in humans [65], and complement activation through the alternative pathway.

Anti-M2e IgG were found to provide antiviral protection by the elimination of infected cells by antibody-dependent cell mediated cytotoxicity (ADCC) or by complement-mediated phagocytosis; natural killer cells, alveolar macrophages and dendritic cells were shown to play an important role [66, 67]. Also, Fc receptors were essential for anti-M2e-mediated immune protection against influenza A virus challenge. Mouse IgG2a/c isotype antibodies possess the strongest effector function and are predominantly involved in ADCC because of their ability to bind all three types of Fc receptors (FcγRI, FcγRIII, FcγRIV) [68]. Mozdzanovska et al. showed that naïve mice protected by passive immunisation with monoclonal anti-M2e antibodies (14C2) isotype G2a experienced lower weight loss and milder symptoms after infection with influenza A virus in comparison with mice protected by the same dose of isotype G1 or G2b anti-M2e antibodies [63, 69]. The protection of mice immunised with M2e-based constructions against lethal influenza challenge was reported to correlate with a high level of IgG2a [59, 63, 67, 70]. In our work, we examined post-immunisation isotype antibody profile (IgG, IgG1 and IgG2a) as well as splenocyte-activated cytokine production (IL-4, IFN-γ). Immunisation of mice with recombinant Flg-2M2eh2M2ek protein led to the generation of specific IgG1 and IgG2a, with predominant IgG1 (IgG1/IgG2a = 10–12), which suggested a Th2-type immune response. These data correlated with the results of the stimulation of immunised mice splenocytes by synthetic М2еh peptide. CD4+ T-cells secreted only IL-4, but not IFN-γ, thus indicated formation of Th2 cells. The type of immune response to flagellin is determined by the protein form—soluble or membrane-anchored. Soluble flagellin (monomeric and polymeric) is known to induce Т- and B-cell immune responses that are specific to flagellin itself and to co-administer the antigen with a strong shift to Th2 type response [38, 70–73], while the membrane-anchored form induces Th1 [70]. Immune response to the flagellin-based recombinant protein also depends on the antigen fused to flagellin [38]. Although the IgG isotype has been found to be critical for the induction of protective immunity after vaccination with M2e-based recombinant proteins, and as the protection is likely correlated with IgG2a antibodies, a high level of IgG1 antibodies can compensate their low ability to activate the effector cells, especially in the case of high epitope density on the target cell [36]. It is known that macrophages contribute to the phagocytosis of influenza virus-infected cells and express FcγRIII [74, 75]. It has been demonstrated that phagocytosis of IgG1-opsonised but not IgG2a-opsonised cells by macrophages depends on FcγRIII [76]. El Bakkouri et al. [67] showed that alveolar macrophages were critical for protection by anti-M2e IgG1 antibodies, providing at least partial protection of mice from lethal influenza infection. Schmitz N. et al. [77] immunised mice with M2e fused to capsid protein of RNA-bacteriophage АР205. Mice immunised with М2е-АР205 with the addition of the TLR7 ligand developed dominant IgG2а/c isotype antibodies, and those immunised without the TLR7 ligand developed dominant IgG1 isotype antibodies. Wherein all vaccinated mice were protected from lethal infection with 4LD_50_ of influenza A/PR/8/34, mice immunised with addition of the TLR7 ligand revealed significantly lower morbidity.

Thus, the elimination of influenza-infected cells by the intranasal administration of M2e-based vaccines may be mediated not only by IgG2a/c but also by IgG1 through FcγRIII activation of alveolar macrophages and by sIgA through FcαμR activation of effector cells or by alternative pathways of complement activation.

A number of studies [6–9, 12–14, 54, 78] have shown the importance of M2e antigen presentation in a multimeric form. Increasing the M2e copy number (up to 3–4 tandem copies) in the recombinant protein lead to a considerably increased M2e-specific response and a higher survival rate of vaccinated animals upon lethal challenge with influenza. Furthermore, the native М2 protein forms a tetramer that contains structural epitopes, playing an important role in immunogenicity. Oligomer-specific antibodies can be induced by the recombinant M2e, which simulates the native structure of the M2 tetramer on the surface of the viral particle [9, 67, 79]. Such antibodies are capable of binding to the surface of cells infected with influenza virus or cells, expressing native tetramer М2. Vaccines that stimulate the formation of antibodies specific not only to linear but also to conformational epitopes possess a higher protective effect. In our Flg-recombinant protein, although four copies of M2e (M2eh-M2ek-M2eh-M2ek) were linked consequentially, post-immunisation antibodies, specific to linear M2e epitopes, were also able to bind the conformational epitopes of native M2 protein expressed on the surface of infected MDCK cells. Moreover, post-immunisation antibodies bound in an equal rate to cells infected with both human influenza virus A/PR/8/34 (H1N1) and avian influenza virus A/Chic ken/Kurgan/05/05 RG (H5N1). Thus, the presence of four copies of two variant M2e within the Flg-based recombinant fusion protein induced the formation of specific antibodies that recognised conformational epitopes of tetramer М2 proteins of influenza A virus strains of different origin.

Our results demonstrated that the immunisation of mice with recombinant protein Flg-2M2eh2M2ek resulted in equal protection against lethal infection with human influenza viruses A/PR/8/34 (H1N1) and А/Aichi/2/68 (H3N2), as well as against the avian influenza virus А/Chicken/Kurgan/05/05 RG (H5N1). Immunised mice expressed lower body weight loss compared to the control group. The protective effect of Flg-2M2eh2M2ek recombinant protein was also confirmed by a significant reduction of viral load (on 1,8–4,0 lgTCID50) in the lungs of immunised mice on day 6 after challenge with influenza viruses А/Chicken/Kurgan/05/05 RG (H5N1), А/Aichi/2/68 (H3N2), A/PR/8/34 (H1N1) and A/Singapore/1/57 (H2N2).

The simultaneous inclusion of M2e of human and avian influenza A viruses in flagellin-based recombinant vaccine led to the induction of systemic and mucosal immune responses to linear and conformational epitopes of the M2 protein ectodomain of both human influenza A viruses and avian influenza virus A/H5N1. A combination of enhanced anti-M2e sIgA and IgG (with different action mechanisms) in the respiratory tract of immunised animals coincided with almost completely protected mice from lethal influenza A challenge. The data obtained in this study show the potential for the development of a candidate influenza vaccine on the basis of heterologous M2e covalently linked to flagellin with broad spectrum protection against influenza A viruses of various origins. There are a variety of influenza viruses having extremely divergent M2e sequences while not all of them have the pandemic potential. Though a threat posed by newly emerging avian influenza A viruses such as H7N9 highlights that protection against these highly divergent viruses needs to be addressed.

## Supporting Information

S1 ARRIVE Checklist (DOCX)

S1 Fig Amino acid sequence of M2 and NA proteins of mouse-adapted A/Aichi/2/68 (H3N2). A. Alignment of M2 protein amino acid sequence of wild-type A/Aichi/2/1968(H3N2) virus (GenBank accession: CY121118) and mouse adapted variant (partial sequence). No amino acid changes detected. M2e peptide is underlined. B. Alignment of neuraminidase amino acid sequence of wild-type A/Aichi/2/1968(H3N2) virus (GenBank accession: CY121117) and mouse adapted variant (partial sequence). No amino acid changes detected.
(TIF)

S1 Table Antigen specificity of mouse-adapted A/Aichi/2/68 (H3N2) in HAI. (PDF)
