# Supplementary material for: Protection against Multiple Influenza A Virus Strains Induced by Candidate Recombinant Vaccine Based on Heterologous M2e Peptides Linked to Flagellin
Source: PLoS One. 2015 Mar 23;10(3):e0119520. doi: 10.1371/journal.pone.0119520 (PMC4370815; doi:10.1371/journal.pone.0119520)
Supplement: S1 ARRIVE Checklist — (DOCX) [file pone.0119520.s001.docx]

**The ARRIVE Guidelines Checklist**

Stepanova L.^1^, Kovaleva A.^1^, Potapchuk M.^1^, Korotkov A.^1^, Kasyanenko M.^1^, Sergeeva M.^1^, Kotlyarov R.^2^, Ravin N.^2^, Tsybalova L.^2^

Similar protection against human and avian influenza A viruses induced by candidate vaccine with consensus human and A/H5N1 M2e on the platform of flagellin

|  | Item |  |
| --- | --- | --- |
| Title | 1 | Similar protection against human and avian influenza A viruses induced by candidate vaccine with consensus human and A/H5N1 M2e on the platform of flagellin |
| Abstract | 2 | BACKGROUND AND PURPOSE: Matrix 2 protein ectodomain (M2e) has been considered as a promising candidate for a broadly spectrum influenza vaccine. But candidate influenza vaccine for human influenza A led to partial protected from avian influenza viruses because of some difference in M2e sequences. In this work we evaluated the possibility of obtaining similar ptotection and immune response on recombinant protein on the basis of flagellin as a carrier for the M2e peptides of human and avian influenza A viruses.  EXPERIMENTAL APPROACH: Female BALB/c mice were immunized intranasally (i.n.) on the days 0 (primary), 14 (first boost), 28 (second boost) with 50 μg/0.1ml (7,5 μg of M2e) of Flg-2M2eh2M2ek. Control mice were injected PBS buffer intranasally in volume 0.1 ml. On days 28 and 42 we determined anti-M2e IgG, IgG1, IgG2a in blood and IgG, IgA in BAL. At 14 day after last immunization proliferative response to M2e peptide in splenocytes was tested. At day 14 post immunization CD4 T-cells in spleen activated with M2e peptide were tested on production of IL-4 and IFN-γ by Intracellular Cytokine Staining (ICS) assay. Mice were challenged intranasally 2 weeks after the final immunization with lethal doses of influenza viruses A/PR/8/34 (H1N1), A/Aichi2/68 (H3N2), A/Kurgan/05/05 RG (H5N1). The animals were monitored daily during 2 weeks for survival, and weight loss.  KEY RESULTS: Intranasal immunization of Balb/c mice with recombinant protein significantly elicited anti-M2e IgG in serum and BAL and sIgA in BAL. Antibodies induced by fusion protein Flg-2M2eh2M2ek bind efficiently to the synthetic peptides corresponding to the consensus human M2e sequence as well as M2e sequence of A/Kurgan/05/05 RG (H5N1) and recognize native M2e epitopes exposed on the surface of the MDCK infected with A/PR/8/34 (H1N1) and A/Kurgan/05/05 RG (H5N1) in a equal rate. Immunization of mice with Flg-2M2eh2M2ek lead to both anti-M2e IgG1 and anti-M2e IgG2a response with IgG1 prevalence. We observed a significant intracellular production of IL-4, but not IFN-γ by CD4+ T-cells in spleen following Flg-2M2eh2M2ek immunization. Immunization with Flg-2M2eh2M2ek fusion protein, containing both M2e consensus human and M2e H5N1, protected mice in similar rate from lethal challenge as with human influenza A viruses (H1N1, H3N2) so with avian influenza virus (A/H5N1), vaccinated animals experienced significantly less weight loss and decreased lung viral titers than the control mice.  CONCLUSION AND IMPLUCATIONS: The data obtained in this study shows the availability the develop candidate influenza vaccine on the base of flagellin with broad spectrum protection from influenza A viruses of distinct origin. |
| INTRODUCTION | | |
| Background | 3 a | The extracellular domain of the influenza virus M2 protein, M2e, is a promising target for the development of recombinant vaccines against influenza (Neirynck et al., 1999; Fiers et al., 2009). However, this peptide is poorly immunogenic when delivered alone and need to be linked to a carrier molecule. Here we use flagellin of *Salmonella typhimurium* as such adjuvant carrier. A number of studies revealed that fusion of multiple copies of M2e to a carrier increase M2e-specifiс immune response (De Filette et al., 2005), particularly, fusion protein containing four consecutive copies of human M2e linked to flagellin protected mice from a lethal challenge with human inﬂuenza A virus (Huleatt et al., 2008). The sequence of M2e is almost universally conserved in influenza strains isolated from humans (Ito et al., 1991; Neirynck et al., 1999) but differs in newly emerging influenza strains of animal origin such as the avian influenza A virus of the H5N1 subtype that caused hundreds of cases of illness and lethal outcomes several years ago, suggesting that a specific M2e-based vaccine against such strains would be required. |
|  | b | For this study we used Balb/c mice as a common object for research of immune response to vaccines and protection against influenza A viruses. |
| Objectives | 4 | Therefore, the objective of this study was to investigate the possibility of obtaining the similar protection and immune response on candidate influenza vaccine on the basis of flagellin as a carrier of the M2e peptides of human and avian influenza A viruses. |
| Ethical statement | 5 | The study was carried out in strict accordance with Russian Guidelines for the Care and Use of Laboratory Animals (1977) and Committee on the Ethics of Animal Experiments of Research Institute of Influenza (Permit Number: 01213). All efforts were made to minimize the suffering of the animals. Mice were housed in cages provisioned with water and standard food and monitored daily for health and condition. More than 25% body weight loss was used as a criterion for early euthanasia. The animals were euthanized by CO2 inhalation for 5 minutes. After final monitoring (14 day post challenge) all the survived mice were humanely euthanized using CO2 inhalation for 5 minutes. |
| Study design | 6a | Six groups of 10 mice each were studied in protection experiments (lethal dose challenge): 1.1 Female BALB/c mice immunized intranasally with Flg-2M2eh2M2ek and challenged with A/Kurgan/05/05 RG (H5N1); 1.2 Female BALB/c mice immunized intranasally with Flg-2M2eh2M2ek and challenged with A/PR/8/34 (H1N1); 1.3 Female BALB/c mice immunized intranasally with Flg-2M2eh2M2ek and challenged with A/Aichi2/68 (H3N2); 1.4Female BALB/c mice administrated intranasally with PBS and challenged with A/Kurgan/05/05 RG (H5N1); 1.5 Female BALB/c mice administrated intranasally with PBS and challenged with A/PR/8/34 (H1N1); 1.6 Female BALB/c mice administrated intranasally with PBS and challenged with A/Aichi2/68 (H3N2).  Eight groups of 5 mice each were studied in experiments on detection of viral titers in lung: 2.1 Female BALB/c mice immunized intranasally with Flg-2M2eh2M2ek and challenged with A/Kurgan/05/05 RG (H5N1); 2.2 Female BALB/c mice immunized intranasally with Flg-2M2eh2M2ek and challenged with A/PR/8/34 (H1N1); 2.3 Female BALB/c mice immunized intranasally with Flg-2M2eh2M2ek and challenged with A/Aichi2/68 (H3N2); 2.4 Female BALB/c mice immunized intranasally with Flg-2M2eh2M2ek and challenged with A/Singapore/1/57 (H2N2); 2.5Female BALB/c mice immunized intranasally with PBS and challenged with A/Kurgan/05/05 RG (H5N1); 2.6 Female BALB/c mice immunized intranasally with PBS and challenged with A/PR/8/34 (H1N1); 2.7 Female BALB/c mice immunized intranasally with PBS and challenged with A/Aichi2/68 (H3N2); 2.8 Female BALB/c mice immunized intranasally with PBS and challenged with A/Singapore/1/57 (H2N2).  Two groups of 5 mice each were studied in immune response experiments: 3.1. Female BALB/c mice immunized intranasally with Flg-2M2eh2M2ek; 3.2 Female BALB/c mice administrated intranasally with PBS. |
|  | b | In all experiments in this study animals were randomized into immunized and control groups without individual marking |
|  | c | In the study, n refers to number of animals, number of samples from each experimental group or individual experimental group for mean body weight estimation. |
|  | d | body weight, survival rate  second boost  priming  challenge  first boost  Days -7_ __0_ 1___________14____________28____________42__________48_______56_  - blood sampling, - BAL sampling, - spleen sampling, - lung sampling  Figure 1. Experimental timelines. Mice were immunized on day 1 (priming), 14 (first boost), 28 (second boost). Two weeks post second boost (42 day) mice were challenge with lethal dose of influenza viruses. The animals were monitored daily during 2 weeks for survival and weight loss. |
| Experimental procedures | 7 a | All mice were immunized and challenged intranasally (0.1 ml of preparation in two nostrils) under inhalation anesthesia (isoflurane 2-3% mixed with 30% oxygen (O2) and 70% nitrous oxide (N2O)). Mice were sacrificed by CO_2_-box for euthanasia for 5 minutes (Vet Tech Solutions) for blood, BAL, spleen, lung sampling. More than 25% body weight loss was used as a criterion for early euthanasia. After final monitoring (14 day post challenge) all the survived mice were humanely euthanized using CO2 inhalation for 5 minutes. |
|  | b | All manipulations with animals (immunization, challenge, sampling) were performed in the light phase (morning) |
|  | c | The animals were tested in home cage |
|  | d | The intranasal route of immunization may be used for the recombinant proteins on the basis of flagellin because flagellin is an effective mucosal adjuvant. |
| Experimental animals | 8 a | Female BALB/c (17.4±1.6), aged 6-8 weeks, were included (n=110). |
|  | b | Female BALB/c mice were obtained from the Institute of Animal Care (Pushchino, Russia) and acclimatized for 7 days. Vendor health reports indicated that mice were free of known viral, bacterial, parasitic pathogens and influenza virus naïve. |
| Housing and husbandry | 9 a | Animals were housed in type 2 cages (10 mice in cage) filled with Lignocel (hygiene animal bedding). |
|  | b | Animals were housed in a 12-houre light/dark cycle, with room temperature (22±1^0^C) and humidity (55±5%) controlled room. All mice were allowed free access to water and maintenance diet containing 1.1% calcium (Chara diet, Assortment agro, Russia). |
|  | c | All animals were monitored daily during experiment for health status. |
| Sample size | 10 a | One hundred and ten mice were used in experiment. Fifty five animals of groups 1.1-1.3, 2.1-2.4, 3.1 immunized intranasally (i.n.) with Flg-2M2eh2M2ek. Fifty five animals of groups 1.4-1.6, 2.5-2.8, 3.2 received intranasally PBS and served as control. |
|  | b | The difference between antibody levels (n=5), index of proliferation (n=3), percent of CD3^+^CD4^+^ IL-2 and IFN-γ producing cells (n=3), viral titers in lung (n=5) was evaluated by Mann-Whitney U-test. Significant differences in survival among mouse groups (n=10/group) were analyzed by Montel-Cox test, difference in body weight loss was evaluated by Wilcoxon test. |
|  | c | - |
| Allocating animals to experimental groups | 11 a | Mice were allocated in research and control groups in a random way, thus ensuring the mean body weight of mice in each group was similar prior to beginning of experiment. |
|  | b | For the protection experiment animals for research and control group were selected in a random way. For the anti-M2e immune response experiment animals from immunized and control groups were selected in a random way. |
| Experimental outcomes | 12 | One primary outcome result was analyzed: titers of anti-M2e IgG (day 28). In addition, three secondary outcome measures were evaluated: anti-M2e (IgG, IgG1, IgG2a, IgA) humoral and cellular immune response (day 42), lung viral titers on 6^th^ day post challenge (day 48), protection of immunized mice from lethal viral challenge (days 42-56). |
| Statistical methods | 13 a | Mann-Whitney U-tests for non-normally distributed data (ELISA results, T-cell immune response results, viral titers) were performed to compare the differences in immunized and control mice. Montel-Cox test was performed to compare the difference in survival rate and Wilcoxon test was performed to compare the difference in mean weight loss between immunized and control mice. |
|  | b | The experimental unit was an individual mice or sample (blood, BAL, spleen, lung) from individual mice or individual experimental group for mean body weight estimation. |
|  | c | - |
| RESULTS | | |
| Baseline data | 14 | The animals’ health status was monitored by a health surveillance programme according to Recommendation of the European Laboratory Animal Science Associations 2001 (FELASA) “Recommendation for health monitoring of rodent and rabbit colonies in breeding and experimental units”. |
| Number analysed | 15 a | Antibody titers were assessed for 5/5 mice in groups 3.1, 3.2. Lung viral titers were assessed for 5/5 mice in groups 2.1-2.8. Eight mice from immunized group (8/10) and one from control (1/10) survived post challenge with А/PR/8/34 (H1N1). Ten mice from immunized group (10/10) and two from control (2/10) survived post challenge with А/Aichi/2/68 (H3N2). Nine mice from immunized group (9/10) and one from control (1/10) survived post challenge with А/Kurgan/5/05 RG (H5N1). |
|  | b | One hundred and ten animals were utilized for this study and 110 were included and completed. |
| Outcomes and estimation | 16 | In accordance with ARRIVE guidelines (Kilkenny et al. 2010) we have reported measures of precision, confidence, and n to provide an indication of significance.  C  Figure 3. Anti-M2e antibody response in serum.  BALB/c mice (n=5/group) were immunized i.n. with 50 μg of Flg-2M2eh2M2ek recombinant protein on days 0, 14, 28. Mice of control group were administered with PBS.  (C) Anti-M2e IgG subclasses tested against M2ek and M2eh in serum 2 weeks post second boost were determined by ELISA. Statistically significant differences between IgG1 and IgG2a levels: p<0.01. Statistical significance was determined using Mann-Whitney U-test. The P values between immunized and control group are indicated. Titers of antiM2e IgG1 ranged to M2eh from 26390 to 45290 (mean=35840), to M2ek ranged from 11334 to 44986 (mean=28160). Titers of antiM2e IgG2a ranged to M2eh from 1777 to 5263 (mean=3520), to M2ek ranged from 1437 to 3043 (mean=2240)  **A B**  Figure 5. M2e specific T-cell response in spleen.  BALB/c mice (n=3/group) were immunized i.n. with 50 μg of Flg-2M2eh2M2ek recombinant protein on days 0, 14, 28. Splenocytes were isolated from 3 mice of each group at day 14 post second boost and assayed for a M2e-stimulated proliferation (A) and M2e specific CD4^+^ T cell response (B). IS ranged in immunized mice from 1,51 to 1,89 (mean=1,69) and in PBS group ranged from 0,79 to 1,35 (mean=1,02). Percent of IL-4+ CD3+CD4+ cells ranged in immunized mice from 0,13 to 0,16 (mean=0,14) and in PBS group ranged from 0,07 to 0,11 (mean=0,09).  Data are presented as the mean±SEM. The index of stimulation (IS) was calculated using the following equation: OD of M2e-treated cells/OD of untreated cells. Statistical significance was determined using Mann-Whitney U-test. The P values between immunized and control group are indicated.  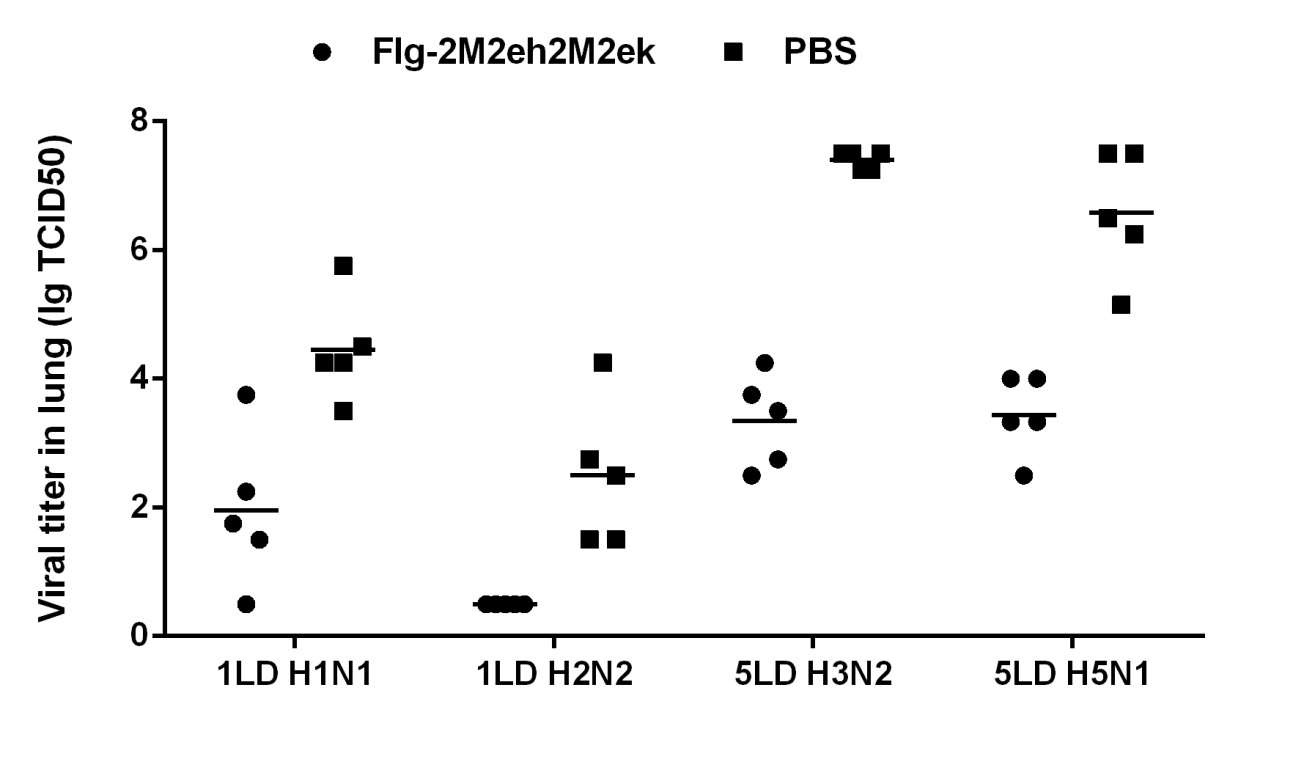  Figure 7. Detection of viral titer in mouse lung.  Mice (n=5/group) immunized with Flg-2M2eh2M2ek fusion peptide were i.n. challenged with 1LD_50_ А/PR/8/34 (H1N1), 1LD_50_ А/Singapore/1/57 (Н2N2), 5LD_50_ А/Aichi/2/68 (H3N2), 5LD_50_ А/Kurgan/5/05 RG (H5N1) and viral titers were detected 6 days post challenge. The data are expressed as lg TCID_50._ Horizontal bars indicate mean among 5 mice per group. The lower limit of detection is 0,5 lg TCID_50._ Statistical significance was determined using Mann-Whitney U-test. The P values between immunized and control group are indicated. Viral titers in lung in immunized mice range from 0.75 to 3.15 (mean=1.95) post challenge with A/H1N1, from 2.64 to 4.06 (mean=3.35) post challenge with A/H3N2, from 0.5 to 0.5 (mean=0.5) post challenge with A/H2N2, from 4.37 to 5.13 (mean=4.75) post challenge with A/H5N1. Viral titers in lung in PBS group range from 4.41 to 4.85 (mean=4.45) post challenge with A/H1N1, from 7.27 to 7.53 (mean=7.4) post challenge with A/H3N2, from 1.5 to 3.5 (mean=2.5) post challenge with A/H2N2, from 5.6 to 7.56 (mean=6.58) post challenge with A/H5N1. |
| Adverse events | 17 a | There were no adverse events after immunization in each experimental group. |
|  | b | There were no modifications to the experimental protocol. |
| DISCUSSION | | |
| Interpretation/ scientific implications | 18 a | The aim of our study was to investigate the possibility of obtaining the similar protection and immune response on candidate influenza vaccine on the basis of flagellin as a carrier of the M2e peptides of human and avian influenza A viruses.The sequence of M2e is almost universally conserved in influenza strains isolated from humans [Ito et al., 1991; Neirynck et al., 1999] but differs in newly emerging influenza strains of animal origin such as the avian influenza A virus of the H5N1 subtype that caused hundreds of cases of illness and lethal outcomes several years ago, suggesting that a specific M2e-based vaccine against such strains would be required. However, peptide M2e is poorly immunogenic when delivered alone and need to be linked to a carrier molecule. Here we use flagellin of *Salmonella typhimurium* as such adjuvant carrier. A number of studies revealed that fusion of multiple copies of M2e to a carrier increase M2e-specifiс immune response [De Filette et al., 2005], particularly, fusion protein containing four consecutive copies of human M2e linked to flagellin protected mice from a lethal challenge with human inﬂuenza A virus [Huleatt et al., 2008]. In our study Immunization with Flg-2M2eh2M2ek fusion protein, containing both M2e consensus human and M2e H5N1, induced equal immune response to M2ek and M2eh and protected mice in similar rate from lethal challenge with human influenza A viruses (H1N1, H2N2, H3N2) so with avian influenza virus (H5N1). |
|  | b | - |
|  | c | - |
| Generalisability/translation | 19 | The study of candidate vaccines in mice is a first step in developing vaccines for human use. |
| Finding | 20 | The research was supported by Russian Ministry of Education and Science under Grant agreement 8292 and the fellowship of the President of Russia for young scientists awarded to R.Y. Kotlyarov. . |
|  |  |  |
