# Supplementary figures and images for: Protection against Multiple Influenza A Virus Strains Induced by Candidate Recombinant Vaccine Based on Heterologous M2e Peptides Linked to Flagellin
Source: PLoS One. 2015 Mar 23;10(3):e0119520. doi: 10.1371/journal.pone.0119520 (PMC4370815; doi:10.1371/journal.pone.0119520)

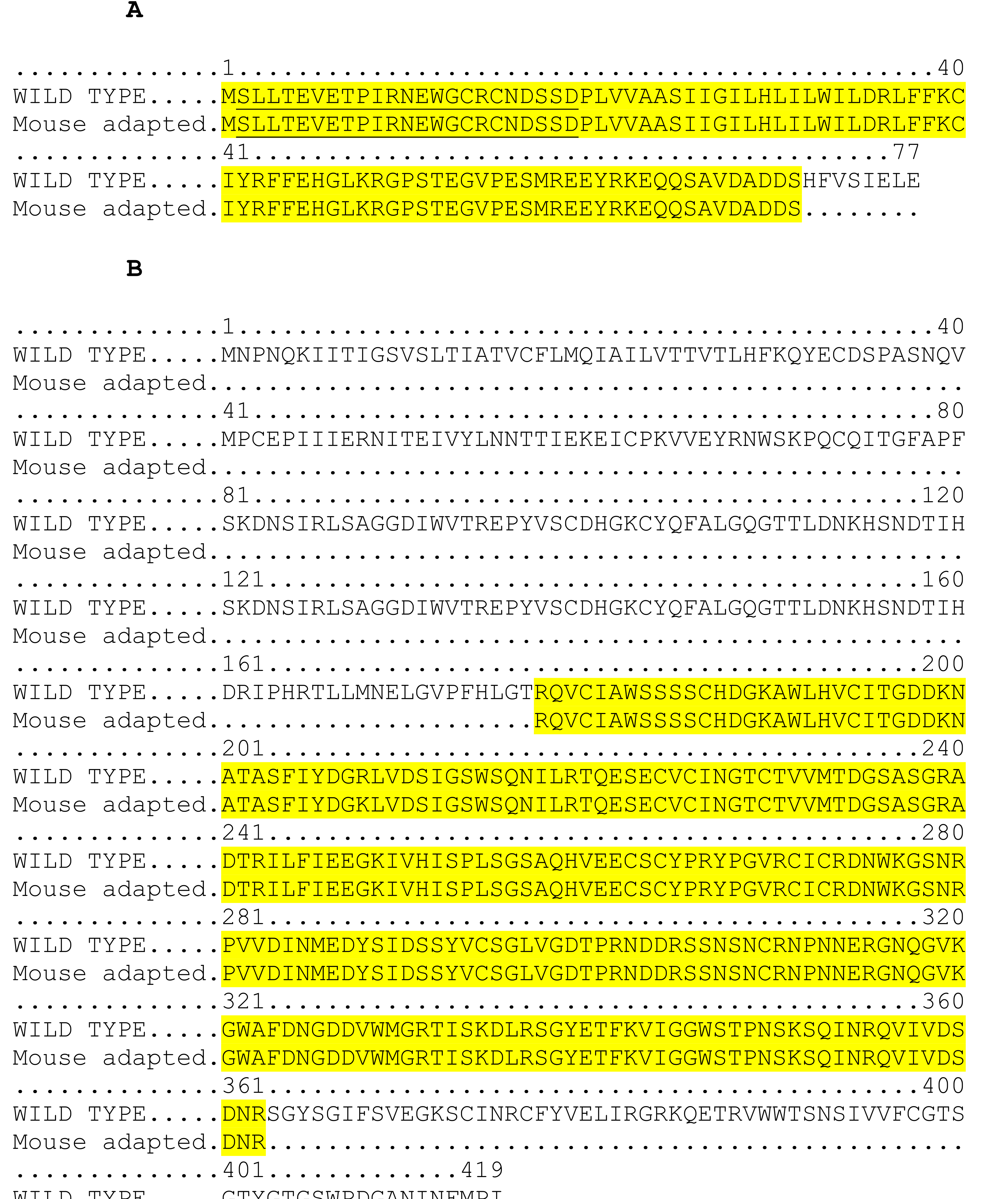

Supplement: S1 Fig — A. Alignment of M2 protein amino acid sequence of wild-type A/Aichi/2/1968(H3N2) virus (GenBank accession: CY121118) and mouse adapted variant (partial sequence). No amino acid changes detected. M2e peptide is underlined. B. Alignment of neuraminidase amino acid sequence of wild-type A/Aichi/2/1968(H3N2) virus (GenBank accession: CY121117) and mouse adapted variant (partial sequence). No amino acid changes detected. (TIF) [file pone.0119520.s002.tif]
