# Supplementary material for: Protection against Multiple Influenza A Virus Strains Induced by Candidate Recombinant Vaccine Based on Heterologous M2e Peptides Linked to Flagellin
Source: PLoS One. 2015 Mar 23;10(3):e0119520. doi: 10.1371/journal.pone.0119520 (PMC4370815; doi:10.1371/journal.pone.0119520)
Supplement: S1 Table — (PDF) [file pone.0119520.s003.pdf]

Diagnostic serum (titres to homologous virus)	Titres in HAI
rat serum to A/Aichi/2/68 (H3N2)<br>(1:320)	1:320
rat serum to A/PR/8/34 (H1N1)<br>(1:320)	<1:10
rat serum to A/Singapore/1/57 (H2N2)<br>(1:320)	<1:10
rabbit serum to A/Kiev/3304/84 (H0N1)<br>(1:320)	<1:10
rabbit serum to A/Victoria/361/11 (H3N2)<br>(1:320)	1:80
rabbit serum to A/California/07/09<br>(H1N1pdm)<br>(1:160)	<1:10
